# A computational approach to rapidly design peptides that detect SARS-CoV-2 surface protein S

**DOI:** 10.1093/nargab/lqac058

**Published:** 2022-08-22

**Authors:** Maryam Hajikarimlou, Mohsen Hooshyar, Mohamed Taha Moutaoufik, Khaled A Aly, Taha Azad, Sarah Takallou, Sasi Jagadeesan, Sadhna Phanse, Kamaledin B Said, Bahram Samanfar, John C Bell, Frank Dehne, Mohan Babu, Ashkan Golshani

**Affiliations:** Ottawa Institute of Systems Biology, University of Ottawa, Health Science Campus, Ottawa, Ontario, Canada; Department of Biology, Carleton University, Ottawa, Ontario, Canada; Ottawa Institute of Systems Biology, University of Ottawa, Health Science Campus, Ottawa, Ontario, Canada; Department of Biology, Carleton University, Ottawa, Ontario, Canada; Department of Biochemistry, Research and Innovation Centre, University of Regina, Regina, Canada; Department of Biochemistry, Research and Innovation Centre, University of Regina, Regina, Canada; The Ottawa Hospital Research Institute 501 Smyth Road, Ottawa, Ontario, Canada; Ottawa Institute of Systems Biology, University of Ottawa, Health Science Campus, Ottawa, Ontario, Canada; Department of Biology, Carleton University, Ottawa, Ontario, Canada; Ottawa Institute of Systems Biology, University of Ottawa, Health Science Campus, Ottawa, Ontario, Canada; Department of Biology, Carleton University, Ottawa, Ontario, Canada; Department of Biochemistry, Research and Innovation Centre, University of Regina, Regina, Canada; Department of Biology, Carleton University, Ottawa, Ontario, Canada; Department of Pathology and Microbiology, College of Medicine, University of Hail, Saudi Arabia; Ottawa Institute of Systems Biology, University of Ottawa, Health Science Campus, Ottawa, Ontario, Canada; Department of Biology, Carleton University, Ottawa, Ontario, Canada; Agriculture and Agri-Food Canada, Ottawa Research and Development Centre (ORDC), Ottawa, Ontario, Canada; The Ottawa Hospital Research Institute 501 Smyth Road, Ottawa, Ontario, Canada; School of Computer Science, Carleton University, Ottawa, Ontario, Canada; Department of Biochemistry, Research and Innovation Centre, University of Regina, Regina, Canada; Ottawa Institute of Systems Biology, University of Ottawa, Health Science Campus, Ottawa, Ontario, Canada; Department of Biology, Carleton University, Ottawa, Ontario, Canada

## Abstract

The coronavirus disease 19 (COVID-19) caused by the severe acute respiratory syndrome coronavirus 2 (SARS-CoV-2) prompted the development of diagnostic and therapeutic frameworks for timely containment of this pandemic. Here, we utilized our non-conventional computational algorithm, InSiPS, to rapidly design and experimentally validate peptides that bind to SARS-CoV-2 spike (S) surface protein. We previously showed that this method can be used to develop peptides against yeast proteins, however, the applicability of this method to design peptides against other proteins has not been investigated. In the current study, we demonstrate that two sets of peptides developed using InSiPS method can detect purified SARS-CoV-2 S protein via ELISA and Surface Plasmon Resonance (SPR) approaches, suggesting the utility of our strategy in real time COVID-19 diagnostics. Mass spectrometry-based salivary peptidomics shortlist top SARS-CoV-2 peptides detected in COVID-19 patients’ saliva, rendering them attractive SARS-CoV-2 diagnostic targets that, when subjected to our computational platform, can streamline the development of potent peptide diagnostics of SARS-CoV-2 variants of concern. Our approach can be rapidly implicated in diagnosing other communicable diseases of immediate threat.

## INTRODUCTION

The rampant spread of COVID-19 on a global scale has become a turning point in the pace of biomedical research, which quickly led to the development of multiple protective immunity vaccines that capitalize on new technologies in record time. On the diagnostic front, the pace of developing reliable and cost-effective SARS-CoV-2 detection methods has been equally remarkable. For instance, detection of viral RNA in nostril or nasopharyngeal swabs by COVID-19 RT-PCR has been the gold standard SARS-CoV-2 nucleic acid amplification-based test (NAATs) in many countries (1,2). COVID-19 RT-PCR assays essentially rely on the amplification of viral RNA, which requires high-quality nucleic acid material (3,4). Although RT-PCR tests remain the gold standard diagnostic tool for SARS-CoV-2 detection, false-positive RT-PCR in the early stages of the COVID era was consistently reported due to contaminated swabs, reagents, amplicons, or other genetic material in the testing site, which collectively amount to ∼4% of false-positive test outputs (5). As the COVID era has progressed with more ongoing refinement of the viral diagnostic tests, another concern has gradually materialized with RT-PCR-based diagnostics, stemming from potential false-negative results associated with the emergence of variants of concern (VOC), with designated oligonucleotides used in these tests potentially unable to recognize the rapidly mutating VOC nucleic acid sequences (6). Accordingly, complementary serological and other rapid tests become a parallel feasible route to consider. Lateral flow devices have thus become available, which are hand-held antigen detection devices that use swabs or saliva samples as inputs (7). However, their sensitivity is low, and they remain challenged with detecting COVID-19 in asymptomatic individuals.

Alternately, the immune reaction has been another diagnostic route that tracks down blood antibodies generated in response to SARS-CoV-2 infection. This is achieved using ELISA-based methods, which can be scaled up in a high throughput fashion to assess thousands of samples in a resource-effective manner (8). However, this approach does not reflect the current infection status of the individual, since antibodies generated against SARS-CoV-2 infection can continue to circulate in the person's blood after treatment. Thus, current advances in ELISA-based approaches have proved to be specific and sensitive as early as three days post infection. But differences still exist in laboratory outcomes and commercially available ELISA tests. For instance, not all antibodies are suitable for labeling and this has been an inherent limitation of this approach (7). Fluorescence immunochromatographic assay (FICA) represents an alternate antibody/antigen recognition strategy that has been developed recently (9). This method is highly sensitive and also enables rapid testing. Although immunological assays are sensitive and highly informative, a high rate of false positives due to cross-reactivity with other antibodies or previous coronavirus infection remains in a lingering outcome. Other serological methods include lateral flow immunochromatographic assay (LFIA), chemiluminescent immunoassay (CLIA), and the neutralization assay (10). LFIA often generates false negative outputs due to high rates of non-specific binding, whereas CLIA requires a longer development time, and also relies on the generation of IgG and IgM in patients at least two weeks post viral infection (10,11). Other methods to generate antibody-like molecules for detection purposes include phage display and systematic evolution of ligands by exponential enrichment (SELEX) (11,12). These can overcome the drawbacks of the time-consuming ELISA assays, and although these methods are faster, they are experimentally challenging and still require notable preparation time (13,14). Computational approaches have thus emerged as powerful options for effective and rapid development of infectious disease diagnostics. They can predict antigenic epitopes of target viral proteins (15) and prioritize diagnostic protein candidates. Computational-based approaches can alternately target viral proteins of interest using short peptide sequences, with high precision, even when the target protein is mixed with dense populations of irrelevant proteins, as described in this study.

Here, we applied our non-conventional computational algorithm, termed In-Silico Protein Synthesizer (InSiPS), to generate a series of peptides that bind to two different regions of the SARS-CoV-2 spike (S) protein. As we previously demonstrated (11), InSiPS utilizes alternative protein–protein interactions (PPIs) to generate short peptide sequences with strong affinity to protein targets. As a proof-of-concept, we showed high binding ability of designed peptides to three yeast proteins, which were confirmed using various assays (11). However, the applicability of this tool to target pharmaceutically important proteins remained unexplored. Due to the rampant spread of SARS-CoV-2 and the growing need to devise sound diagnostic and therapeutic strategies for rapid containment of this global pandemic, we thought of utilizing our InSiPS algorithm to generate peptide sequences that target biomedically important and surface displayed SARS-CoV-2 spike (S) protein target. Unlike other computational methods that rely on 3D structural pockets of target proteins, InSiPS is conceptually built on the principle of alternative PPIs, mediated by small co-occurring motifs (12–14,16). Our algorithm designs and subsequently prioritizes interacting peptides (IPs) from a random pool of hundreds of thousands of peptide sequences over numerous generations of mutations and crossovers, enabling it to analyze billions of peptide affinities to target proteins in less than 2 weeks.

To this end, we designed short peptide sequences that target either the receptor binding domain (RBD) of SARS-CoV-2 S protein, or the S1/S2 region, since both regions are directly implicated in the early stages of viral entry into host cells (17). The top scoring five peptides recognizing RBD, and five additional peptides targeting the S1/S2 region were subjected to ELISA and Surface Plasmon (SPR) approaches for binding assessment of their cognate targets. Also, salivary peptidomics performed on COVID-19 patients allowed to prioritize a set of SARS-CoV-2 hits that can be used towards COVID-19 peptide diagnostics.

## MATERIALS AND METHODS

### Computational peptide design

To design peptides with specific binding profiles, the InSiPS algorithm was utilized as described before (16) with certain modifications. The human PPIs reported on or before May 1st, 2020 was downloaded from the BioGRID database, while the human cell surface proteins downloaded from Cell Surface Protein Atlas (18) was used as a negative set for counter selection. The length of the initial pool of random synthetic sequences was set at ∼40 amino acids. This achieves sufficient target specificity while maintaining cost-effectiveness when select peptides are synthesized for experimental validations. A processor cluster containing >10 000 processor cores was utilized to evaluate synthetic sequences for their interaction profiles against the target protein and the negative set, as in (19). For each sequence, the likelihood of interaction with the target and non-target proteins was predicted. This in turn determines a fitness value for each candidate. As a general rule, a positive interaction score is set at a specificity of 99% resulting in a sensitivity of ∼35%. This was performed using the Protein Interaction Prediction Engine (PIPE) algorithm (19,20). The interaction profile for each of the sequences estimating a likelihood of an interaction with the target vs. non-targets was evaluated. The resulting prediction scores are combined into a single score, fulfilling target binding and penalizing predicted off-target interactions. The fitness of a given sequence was calculated as follows: Fitness(seq) = [1 – MAX(PIPE(seq, non-targets)) × PIPE (seq, target)]

Based on the calculated fitness values, the genetic algorithm was then built on a new set of sequences using selection, copy, mutation and cross-over operations. This process is iterated until termination criteria are met. The mutations and cross-over rates were set as before (16). A typical interaction landscape for a sequence generated in this manner is shown (Supplementary Figure S1). The objective of the genetic algorithm is to optimize the fitness values of the candidates and find a protein sequence with the desired properties. This typically takes between 500 and 1000 iterations of the genetic algorithm. Since the computational needs are massive, a typical run requires ∼6–8 days of computation time using 10 000 processor cores.

### Peptide synthesis and purification

Amino acid sequences corresponding to either FLAG (DYKDDDDK) or myc (EQKLISEEDL) tag as well as 6x-His were added to gene-synthesized peptide sequences, cloned into a pET22 expression vector under the control of a T7 promoter. The constructs were then transformed into *Escherichia coli*, and the peptides were affinity purified from *E. coli* cell lysates to evaluate their binding affinity to S protein target through ELISA assay. The designed peptides were subsequently validated by SPR assay upon commercial synthesis using 95% pure peptide synthesized at 5 mg by Shanghai Royobiotech®.

### ELISA analysis

ELISA plates with immobilized protein S or RBD and COVID-19 ELISA kits were purchased from BioLegend^®^. ELISA plates with immobilized anti-FLAG antibodies and non-immobilized plates were purchased from Thermo Fisher Scientific^®^. The antibody against recombinant SARS-CoV-2 spike protein S1 (HC2001) was purchased from GenScript^®^ and anti-myc/c-myc antibody was from SantaCruz^®^.

To confirm and quantify the binding efficiency of designed peptides to the S protein, RBD, or PBS (as a control) sandwiched-like ELISA was performed using immobilized S, RBD, and PBS plates. The plates were incubated with designed peptides (2 μg/ml) for 1 h. After several washes, the wells were incubated with anti-FLAG antibody for 1 h at room temperature. Avidin-HRP was then added followed by substrate and stop solution for colorimetric quantification after 10-15 min. Commercial HPR-anti-RBD antibody was used as a positive control. In a similar approach, anti-FLAG antibody coated plates were used to immobilize the designed peptides (containing FLAG tags) to the wells. After 1 h of incubation with designed peptides, the wells were washed a few times to remove excess peptides. The plates were incubated with 10 ng/ml cell lysate mixture (human bronchial epithelial cells) plus 1 ng/ml of the S protein for 1 h at room temperature and washed several times. Commercial HPR-anti-RBD antibody was used to detect the bound S proteins.

To capture and detect the S protein in a sandwich ELISA, anti-FLAG antibody coated plates were immobilized with the designed peptide (FLAG-tagged SS2 recognizing the S1/S2 region) to the wells. After incubation and several washes, the plates were incubated with 10 ng/ml of human cell lysate or cell lysate containing varying concentrations of the S protein (0.01–10 ng/ml) for 1 h. After several washes, myc-tagged R1 peptide was added to the plate and incubated for an additional 1 h followed by several washes. HPR-anti-myc antibody was then used for the detection of myc-tagged R1 peptide.

### Study participants and whole saliva supernatant collection

The saliva samples were collected within 3 days from positively confirmed five SARS-CoV-2 and three Alpha VOC COVID-19 individuals, who provided their informed consent to participate in the study approved by the Saskatchewan Health Authority (RED-20–56) and the University of Regina Research Ethics Board. The presence of SARS-CoV-2 and Alpha VOC in COVID-19 individuals was verified using real-time reverse transcription polymerase chain reaction (rRT-PCR) test and whole genome sequencing, respectively, using nasopharyngeal swab sample collection method (21). The participants were provided with sugar-free chewing gum to stimulate saliva, which is instantly collected in a 10 ml screw capped sterile polypropylene collection tube. The tubes containing the saliva samples were then placed on ice and transported to the laboratory, where the virus is heat inactivated at 60°C for 15 min. The resulting saliva sample is transferred to a fresh tube and centrifuged at 8000 × g for 20 min at 4°C to separate the whole saliva supernatant, which is either immediately used or stored at -80°C until further use.

### SPR and salivary preparation for mass spectrometry (MS)

SPR was conducted using an OpenSPR Rev4 instrument (Nicoya Lifesciences) to study interactions between an affinity purified SARS-CoV-2 S protein receptor binding domain (Spike-RBD, residues 333–529; gift from Dr. Joanne Lemieux's group, University of Alberta, Canada), immobilized onto an NTA sensor chip and our InSiPS-generated putative COVID-19 diagnostic peptides at different concentration (5, 10, 15 and 20 μM) at a flow rate of 40 μL/min in running buffer (10 mM HEPES, pH 7.5, 150 mM NaCl, 3 mM EDTA, 0.005% (w/v) P20 surfactant), with adequate injection time (600 s) allowed for interaction to reach steady-state. All SPR experiments were performed in triplicates at 20°C. From the results of the sensorgrams, response units at steady state were plotted against respective ligand concentrations to generate the binding curve. The *K*_d_ was calculated using Nicoya’s TraceDrawer kinetic analysis software.

For MS analysis, from the whole saliva supernatant of SARS-CoV-2 or Alpha VOC COVID-19 individuals, 500 μl sample was filtered in a microcentrifuge tube with a 10-kDa molecular weight cut-off membrane (Millipore Sigma). The resulting peptide fraction was dried and then subjected to mass spectrometry.

### Liquid chromatography–tandem MS analysis

Chromatographic separation of salivary peptides was performed on a Proxeon EASY nLC (Nano-flow liquid chromatography) 1000 equipped with a Thermo Scientific™ Acclaim™ PepMap™ C18 column (50 μm ID × 15 cm, 3 μm particle size, 100 Å pore size), along with water/acetonitrile/0.1% formic acid gradient. About 5 μl of the samples were loaded onto the column for 100 min at a flow rate of 0.30 μl/min. Peptides were separated using 1% acetonitrile and increasing to 3% acetonitrile in the first 2 min and then using a linear gradient from 3 to 24% of acetonitrile for 170 min, followed by gradient from 24 to 100% of acetonitrile for 29 min and wash 10 min at 100% of acetonitrile. Eluted peptides were directly sprayed into mass spectrometer using positive electrospray ionization (ESI) at an ion source temperature of 250°C and an ionspray voltage of 2.1 kV. Full-scan MS spectra (*m*/*z* 350–2000) were acquired in the Orbitrap Elite at 60 000 (*m*/*z* 400) resolution. The automatic gain control settings were 1e6 for full FTMS scans and 5e4 for MS/MS scans. Fragmentation was performed with collision-induced dissociation (CID) in the linear ion trap when ion intensity was set >1500 counts. The 15 most intense ions were isolated for ion trap CID with charge states ≥2 and sequentially isolated for fragmentation using the normalized collision energy set at 35%, activation Q at 0.250 and an activation time of 10 ms. Ions selected for MS/MS were excluded for 30 s. Calibration was performed externally with Pierce LTQ Velos ESI Positive Ion Calibration Solution (Thermo Fisher Scientific, catalog number 88322). The Orbitrap Elite mass spectrometer was operated with Thermo XCalibur software. All samples were analyzed in biological triplicates.

### Peptide identification

The acquired MS spectra data of the patient samples were searched against the reference SARS-CoV-2 target and decoy protein sequences downloaded from the UniProt. In the case of VOC samples, SARS-CoV-2 S protein sequence was replaced with Alpha VOC S protein sequence. Enzyme-free search of 10 kDa peptides was conducted using the SEQUEST (ver. 27, rev. 9) search algorithm. Search parameters were set to a precursor mass tolerance ranging from 2 to 4 Da (daughter mass ion tolerance set to 0 as default value), variable modification of methionine oxidation, protein N-terminal acetylation, and one fixed modification of cysteine carbamidomethylation. Selection criteria of XCorr ≥ 2.0 and DCn ≥ 0.1 were used to select the existence of a SARS-CoV-2 and Alpha VOC peptides within the sample. Using the above stringent criteria on 11,615 peptides identified across all samples, we selected 100 peptides for further analysis (Supplementary Table S1).

### Structural modeling and molecular docking

The de novo protein trRosetta tool was used to predict the unresolved structure of the representative R1 peptide, and the SARS-CoV-2 S protein trimer structure (PDB: 6VWB) was used in this study. Molecular docking study was conducted using Z-Dock (http://zdock.umassmed.edu/), a fast Fourier transform-based algorithm to generate candidate docked structures from rigid protein units. For the high scoring docked structure, residues at the complex interface were determined with LigPlot + 7, as well as protein and ligand interactions were analyzed and visualized via PyMOL.

## RESULTS

**Figure 1.**
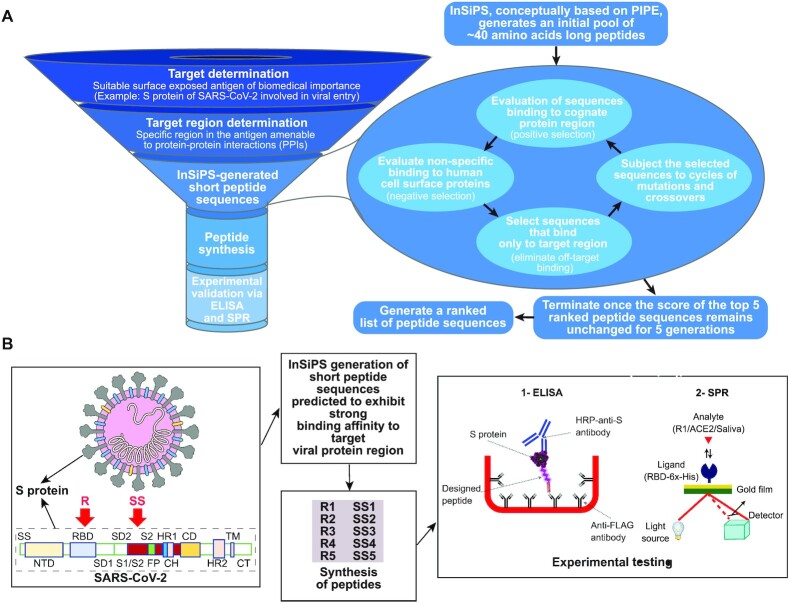
Schematic of the InSiPS strategy used in the current study and its timeline. (**A**) The overall approach for the design and development of peptides that bind to specific protein targets. (**B**) The methodology used to design and develop peptides that bind to two different regions (RBD and S1/S2) of the SARS-CoV-2 spike surface protein S.

The rapid spread of COVID-19 has illuminated the need to develop fast, efficient, and affordable methods to detect emerging pathogens. We sought to utilize our InSiPS algorithm to design peptides that bind to the SARS-CoV-2 surface spike protein S in a time and cost-effective manner. We have previously used this method to develop peptides against yeast proteins; the efficacy of InSiPS to develop peptides against proteins from other organisms, however, has not been examined. The overall strategy (Figure 1A) is composed of four steps that start with target protein determination. These are often proteins exposed on the viral surface, rendering them attractive for detection purposes. Secondly, target region determination, aims to identify the suitable region(s) of the target protein for interacting peptide development. Target region(s) are ideally amenable to PPIs, rendering their amino acid sequences feasibly targeted by synthetic peptides. The last two steps pertain to peptide synthesis for experimental validation. Here, the S protein responsible for SARS-CoV-2 entry into the human host cells was selected as the target. It is the main antigen present on the surface of the viral particles, frequently studied for neutralizing antibodies and vaccine developments (22,23), and globally considered as an effective target for detection purposes (4,24). The S protein recognizes and interacts with the human cell surface angiotensin-converting enzyme 2, ACE2, receptor. This interaction is mediated by the RBD region, a 194 amino acids stretch (aa 331–aa 524) found in S protein (25,26), suggesting surface exposure of this region for interaction with other proteins. RBD is already a common target for therapeutic developments against SARS-CoV-2 (22,23,27,28).

The SARS-CoV-2 S protein additionally contains an important S1/S2 region implicated in viral entry into the host cell upon proteolytic cleavage by host cell proteases (17). After an interaction between RBD and the ACE2 receptor is formed, the S1/S2 region (aa 672–aa 709) is cleaved by the human TMPRSS2 protease. Cleavage of the S protein into S1 and S2 subunits mediates entry of the virus particles into the host cells. The interaction between the S1/S2 region and the host TMPRSS2 suggests that the S1/S2 region may potentially interact with other proteins. We, therefore, selected both the RBD and S1/S2 cleavage sites as target regions for the design of our interacting peptides. The computational design of interacting peptides was set to generate a pool of 20-40 amino acid long peptides with randomly incorporated amino acid sequences. This length of generated peptides satisfies the balance between sufficient specificity in target detection and also the cost-effectiveness of peptide syntheses for downstream experimental verifications and subsequent commercialization attempts. The generated peptide sequences are then evaluated for their potential to interact with RBD or S1/S2 region (positive selection) based on co-occurring interacting motifs. The sequences are also evaluated for their potential to non-specifically interact with human surface proteins (negative selection). Sequences that meet the interaction threshold with the target region (RBD or S1/S2 region) and not the human surface proteins are retained, while the rest of the sequences are eliminated. The retained sequences are then copied and subjected to mutations and crossover events at predefined rates. Sequences generated in this manner are entered into the next round to evaluate their potential interaction with the target region as well as the human surface proteins (negative selection). The procedure is repeated for 1000 cycles or until the interaction score of the top 5 sequences remain unchanged for five generations (Figure 1A). At this point, a list of five sequences ranked for their putative tendency to interact with the target protein (RBD), and 5 other sequences that likely interact with the S1/S2 region, is generated (Figure 1B). InSiPS is conceptually designed on an underlying Protein-Protein Interaction Prediction Engine (PIPE) (12) that generates peptide sequences with strong affinity to target queries. It prioritizes short amino acid sequences that can recognize protein targets guided by PPI records available in public repositories. PIPE analyzes the amino acid sequences of pairs of interacting proteins in literature for the co-occurrence of well-defined motifs. The presence of these motifs has been used as a determining factor for the ability of two proteins to interact (13,14). As the databases of PPIs continue to grow, expansion and refining of these stored sequences become an ongoing process (29). In InSiPS, when queries are fed into the algorithm, InSiPS prioritizes, through many cycles of assessment and refinement as previously explained, subsets of amino acid sequences that can recognize and interact with the query protein sequences (12).

**Figure 2.**
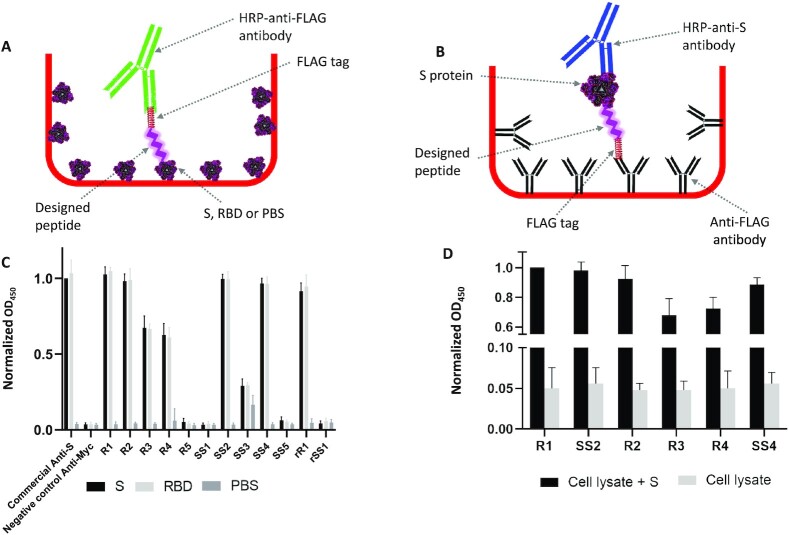
ELISA-like assays to evaluate the binding of the designed peptides to their targets. (**A**) and (**B**) show a schematic of the binding assays used. (A) Target proteins are immobilized to the ELISA wells, whereas in (B) the peptides are attached to the wells by binding to immobilized anti-FLAG antibodies. The affinity of the designed peptides to the immobilized S protein, RBD, and PBS (used as a control) is shown in (**C**). Values are normalized to that obtained by a commercial anti-S antibody (OD_450_). The tendency of the immobilized designed peptides to capture the S protein from a mixture of cell lysates plus the S protein is represented in (**D**). Values are normalized to that obtained by the R1 peptide for the detection of S protein in a mixture of cell lysates and the S protein. Each experiment is repeated at least four times independently.

The top 5 peptides in each group with the highest binding scores to the RBD or S1/S2 region were gene-synthesized by cloning into a pET22 expression vector under the control of a T7 promoter and fused to an N-terminal 6x-His-tag and a C-terminal FLAG- or myc-tag. The constructs were then transformed into *E. coli*, and the peptides were affinity purified from *E. coli*, cell lysates to evaluate their binding capacity to cognate targets using an ELISA-based assay. For this purpose, ELISA plates were coated with RBD, S protein, or BSA (as a control). S protein coated plates contain both the RBD and S1/S2 regions, whereas the RBD plates contain only the RBD region of the S protein. Peptides that interact with the immobilized RBD, S protein, or BSA were detected using an anti-FLAG antibody, with commercial anti-RBD antibody used as a positive control (Figure 2A, B). Of the 5 RBD targeting peptides (R1–R5), 4 generated strong signals (R1–R4) for interaction with both RBD and the S protein (Figure 2C). For the S1/S2 region targeting peptides, two (SS2 and SS4) of the five tested peptides (SS1–SS5) generated strong signals in S-coated wells. These data suggest that a subset of the designed peptides has robustly detected the immobilized S protein in an ELISA-like assay. Peptides can also be commercially synthesized in a timely manner, which may not be cost-effective for long peptides. To illustrate that peptide production methods can be interchangeably used to accompany InSiPS, we also used commercially synthesized peptides in the aforementioned assay and observed similar results (data not shown).

**Figure 3.**
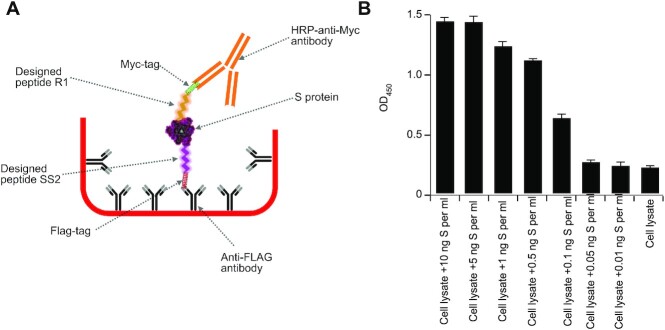
Designed peptides specifically bind to the S protein in sandwich ELISA. (**A**) Schematic of a sandwich ELISA where the designed peptides are used to both capture (SS2 peptide recognize the S1/S2 region) and detect (R1 peptide recognize the RBD region) the S protein from a mixture with cell lysates. Represented in (**B**) the sandwich ELISA can detect the presence of the S protein in the cell lysate mixtures down to 100 pg per ml (0.1 ng/ml). Each experiment is repeated at least four times, independently.

To complement the aforesaid observations, and for cost-effective purposes, one representative peptide from each group as outlined above (R1 recognizing the RBD and SS2 recognizing the S1/S2 region) was additionally subjected to a parallel experiment, but this time by attaching the peptides to ELISA wells and performing a sandwich ELISA-like assay. The two peptides were attached to the wells through immobilized anti-FLAG antibodies and used to capture the S protein from a mixture of cell lysates plus the S protein while using an anti-RBD antibody for detection purposes. Both peptides could capture the S protein (Figure 2D). The pairing of designed peptides against RBD with anti-RBD IgGs in detecting the S protein suggests that these molecules are not mutually exclusive. While both target the RBD region, they recognize different sites within the 194 amino acid long region. Next, we examined whether the designed peptides can be used as both the capture and detection antibody-like molecules in sandwich ELISA assays. To address this, we immobilized the designed peptide SS2, which recognizes the S1/S2 domain of the S protein to the ELISA wells, as above, to capture the S protein, and used a myc-tagged version of peptide R1 for detection purposes (Figure 3A). We observed that without the need for a commercial anti-S antibody, the designed peptide can exclusively capture and detect the S protein within a sandwich ELISA assay (Figure 3B).

**Figure 4.**
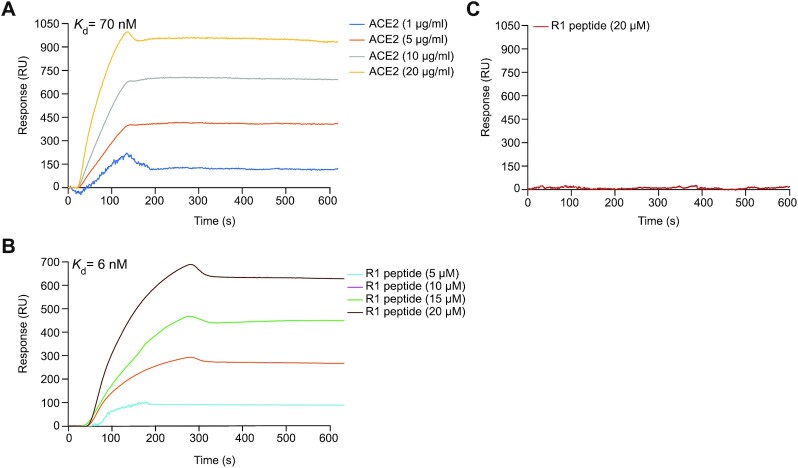
R1 peptide exhibits a strong binding affinity to S protein RBD. (**A**) Spike-RBD (20 μg/ml) was immobilized onto the NTA sensor chip by nickel coupling using 20 mM NiCl_2_, and the ACE2 human receptor was injected at different concentrations (5, 10, 15 and 20 μg/ml) as positive control. (**B**) Affinity purified RBD (20 μg/ml) is immobilized onto carboxyl sensor chip, and R1 peptide was injected at different concentrations (5, 10, 15 and 20 μM) through the SPR flow cell. (**C**) ACE2 (20 μg/ml) was immobilized onto the sensor chip, and R1 was injected at 20 μM concentration as negative control.

To orthogonally validate our data, we estimated the binding affinity of R1 to the RBD region of the S protein using SPR analysis. SPR is a reliable tool for assessing protein interactions in a fast and efficient manner. Due to its high specificity and sensitivity, SPR sensors have been used for the efficient detection of clinical samples in real time (30). As a positive control, the *K*_d_ value of ACE2-RBD interaction was determined to be ∼70 nM (Figure 4A). In addition, the *K*_d_ value for R1-RBD interaction under the same condition was 6 nM (Figure 4B). Similar to our ELISA-based approaches, R1 binding to RBD was specific, as evidenced by no detectable SPR signal when R1 was passed in the flow cell when the human ACE2 was immobilized on the sensor chip as negative control (Figure 4C). Structural prediction of the R1 peptide reveals four beta-strands connected by short loops (Supplementary Figure S2A). Molecular docking of R1 suggests its recognition of the RBD chain A and B interface within the trimeric context of the S protein assembled during SARS-CoV-2 infection (Supplementary Figure S2A). Our analysis also reveals 5 key residues within R1 deemed critical for interaction with the RBD chains A and B (Supplementary Figure S2B).

### Patient-derived salivary peptidomics identify additional priority SARS-CoV-2 diagnostic peptides

The InSiPS-generated peptides outlined above have been developed and prioritized based on established knowledge on SARS-CoV-2 viral entry mechanisms, which require S protein interaction with the human receptor, ACE2. We have thus exploited this information to generate peptide sequences that target the S protein RBD as explained above. In addition, we sought to adopt another patient-driven peptidomics approach to identify the entire pool of SARS-CoV-2 peptides existing in patient saliva (31), which can also serve as another strategy for COVID-19 peptide diagnostics development. To this end, salivary samples collected from 5 patients infected with the wild-type SARS-CoV-2, and 3 patients infected with the Alpha VOC were processed for MS analysis. A snapshot of the top 100 peptides that passed an XCorr ≥ 2.0 and DCn ≥ 0.1 thresholds is provided in Supplementary Table S1. SARS-CoV-2 identified peptides in patient saliva exhibited varied lengths, ranging from 5 to 57 amino acid residues each, with the majority of peptides ranging from 15 to 45 amino acids for both, the Alpha variant and its wild-type counterpart (Supplementary Figure S3A, B).

Importantly, the ∼30-kb SARS-CoV-2 genome encodes 14 open-reading frames (ORFs). ORF1a or its ORF1ab variation encodes polyproteins that are proteolytically processed, resulting in 16 non-structural proteins (NSPs), which collectively assemble the SARS-CoV-2 replicase-transcriptase complex (Supplementary Figure S3C) (32). Of note, NSP5 is the main protease (Mpro), which is a critical component of the replicase-transcriptase complex. It is the main enzyme that processes aforesaid polyproteins by creating multiple cleavage sites, thereby contributing to SARS-CoV-2 maturation (33). In contrast, the 3’ end of the SARS-CoV-2 genome encodes 13 additional ORFs (Supplementary Figure S3C) that encode the viral S protein, 3 other structural proteins, and 9 accessory factors (32). Our salivary peptidomics approach led to the detection of a myriad of SARS-CoV-2 peptides derived from most viral proteins (Supplementary Figure S3C), which has been similarly observed in other viral infections (31). The most repetitively identified peptides, whether in the wild-type SARS-CoV-2 or Alpha VOC patient saliva, belong to NSP5 (Supplementary Figure S3C), suggesting that NSP5-derived peptides may serve as promising COVID-19 diagnostics. This can be achieved via two routes, first by using NSP5-detected peptides from patient saliva as a proxy to develop monoclonal antibody-based diagnostics for rapid usage at ports of entry and community gatherings as SARS-CoV-2 detection tools. Alternately, these NSP5-derived peptide sequences, or other priority peptides detected in patient saliva, can be ‘fed’ into our InSiPS algorithm. Similar to our experimentally demonstrated high affinity peptide diagnostics outlined above, InSiPS can generate novel and potent peptide sequences that strongly target NSP5 regions identified in patient saliva for the subsequent development of antibody-based diagnostics.

A representatively overlapping NSP5-derived peptide region identified in both the Alpha VOC and the wild-type SARS-CoV-2 strain infected patients includes a 6 amino acid stretch (P9-G15) residing in the dimer interface of NSP5 (Supplementary Figure S4A, B). This information serves as a proxy for InSiPS design of novel peptides targeting core NSP5 regions present in patient saliva, with proper overhangs. More importantly and in the case of the P9-G15 NSP5 stretch, and since this stretch localizes to the dimer interface of NSP5, InSiPS can generate disruptive peptide inhibitors that target this region, offering another promising angle on the diagnostic and therapeutic front, which is beyond the scope of the currently presented work.

## DISCUSSION

Different computational approaches have been utilized to design and/or discover peptides and proteins that bind to SARS-CoV-2 proteins. These methods have inherent advantages and disadvantages. They generally rely on the availability of the structural coordinates highlighting the interface residues mediating viral protein interaction with its host protein counterpart. Cao *et al.* (34) computationally generated scaffolds against both the viral S protein RBD and its corresponding helix binding site on the human ACE2 protein. They utilized a Rosetta-based protein builder and a modified protein docking approach to design their peptides. Both approaches rely on the 3D structure of proteins, and scaffold sequences were optimized for target binding and stability, resulting in peptide designs with binding affinities in pico to nanomolar range. They observed that some of their designs blocked SARS-CoV-2 infection of Vero E6 cells. However, the specificities of their scaffolds were not investigated. The S binding site on the ACE2 receptor has also been used as a proxy to design binding peptides and proteins that target S protein with higher affinity than ACE2 (35). A computational approach was utilized to increase the binding affinity of residues that did not appear to engage in favorable interactions with RBD. The predicted affinity of the designed peptides to S protein was higher than ACE2 (35). Computational mutagenesis has also been used to increase the affinity of ACE2 to S protein. The top protein designed in this manner had a reported binding affinity of 1.8 nM (36). Advanced molecular docking approaches that utilize detailed 3D structure of target protein have also been implemented to design peptides targeting the S protein. Recently, Squeglia *et al.* (37) combined molecular docking with single point mutations and molecular dynamics to design high affinity S protein binder, where a series of peptides against the S protein had a binding affinity in the range of 29–200 nM.

A key difference between these approaches and the InSiPS method employed in the current study is that InSiPS uses the primary sequence of the target protein and operates independent of the availability of a detailed 3D structure of the target or its interacting protein partner/substrate. In addition, InSiPS has a built-in function to avoid designing peptides that might interact with a set of undesired proteins. Thus, the generated peptides are designed to offer increased specificity. A drawback for InSiPS can stem from the sites on the target protein against which peptides are designed and interact with. Unlike the approaches that mimic host substrates, peptides created by InSiPS may not hold their potency as the viral protein evolves. Also, since InSiPS uses the database of PPIs to design interacting peptides, its accuracy is correlated to the availability of high confidence PPI data for that particular organism. In the case of S protein examined here, it appears that high affinity binding peptides can be created using certain 3D-based methods (34).

The experimental strategies presented in this work open a new avenue of streamlined COVID-19 peptide diagnostics that combines our InSiPS tool with salivary peptidomics in a manner that can be experimentally verified. Our efforts align with the current objective of rapidly developing reliable and easy-to-use diagnostics of new and emerging communicable diseases, which has gained significant momentum with the eruption of the COVID-19 era. This study outlines the systematic design, synthesis, and experimental evaluation of five peptides developed against each of the RBD and S1/S2 regions of the SARS-CoV-2 S protein. Of the 10 designed peptides, 6 peptides showed promising binding profiles as indicated by ELISA-based assays, suggesting a relative success rate of ∼60%. The tendency of a subset of these peptides to detect remarkably low quantities of the S protein in cell lysate mixtures (as low as 100 pg/ml), in addition to the speed (<2 weeks versus months for the monoclonal antibody-based counterpart) and the low cost (negligible vs. thousands of dollars) of our peptide design and development, collectively render our approach attractive for the streamlined development of COVID-19 peptide diagnostics. In addition, our observations provide evidence for the applicability of InSiPS to design peptides against non-yeast proteins.

The utility of our SARS-CoV-2 detection peptides stems from their capacity to be commercialized for recognizing cognate SARS-CoV-2 targets in patient saliva, which constitutes a rapid, effective, and non-invasive approach for screening potential SARS-CoV-2 carriers in large gatherings or ports of entry. We also surveyed SARS-CoV-2 protein profiles in three patients’ saliva samples using MS analysis, and a number of viral protein fragments exist in high abundance. SARS-CoV-2 identified peptides in patient saliva exhibited varied lengths, ranging from 5 to 57 amino acid residues each, with the majority of peptides ranging from 15 to 45 amino acids for both, the Alpha VOC and its SARS-CoV-2 counterpart (Supplementary Figure S3A, B). The most repetitively identified peptides, whether in the SARS-CoV-2 wild-type strain or its Alpha VOC counterpart in patient saliva belong to the non-structural protein main protease (NSP5) (Supplementary Figure S3C), suggesting that NSP5-derived peptides may serve as promising COVID-19 diagnostics. In the future, it would be of great interest to design peptides that target NSP5 and other high abundance protein fragments and evaluate their usefulness for diagnostic purposes.

It is still unclear whether the approach presented here applies to all or the majority of protein targets. It has previously been documented that certain proteins may not be detected using co-occurring small motifs (19,20). It is possible that such proteins when used as targets may not be as amenable to the approach presented in the current study. Additional investigations are warranted to evaluate the applicability of this method for the effective design of peptides against a wide range of protein targets. However, other protein targets belonging to the same pathogen can be subjected to our approach, rending it widely implicated in detecting different pathogens of immediate threat, upon carefully choosing the target antigen of interest. Although the number of peptides assessed in this study is low, they may point to differences in the ability of the approach to successfully tackle different regions within the same protein. It is expected that certain regions of proteins might be more accessible to interactions than others. The high-resolution cryo-electron microscopy structure of the S protein reveals that the RBD domain seems more accessible than the S1/S2 region and exists within a solvent-exposed surface of the S protein (25). Our observations reported in the current study seem to point to RBD being a better target for designing binding peptides. Future research is needed to better characterize the suitability of different protein regions for peptide development.

Here, we, therefore, present a fast and cost-effective method to computationally design peptides that bind to a protein target of biomedical importance and further illustrate the utility of our approach in SARS-CoV-2 and VOC diagnostics. The binding peptides to SARS-CoV-2 surface protein S were computationally generated based on alternative PPIs mediated by short co-occurring motifs. In the current study, the usefulness of the peptides in ELISA and SPR analyses is demonstrated. It is reasonable to expect that some of the peptides developed in this manner may also be used for other purposes including neutralization, immunoprecipitation, and therapeutic purposes. Due to their small size, they might serve as attractive guide molecules to be combined with other compounds, such as drugs (both small molecules and therapeutic proteins) to form peptide-drug conjugates. In the future, we plan to further examine the usefulness of binding peptides for the detection of a range of protein targets.

## Supplementary Material

lqac058_Supplemental_Files
