# Supplementary material for: A computational approach to rapidly design peptides that detect SARS-CoV-2 surface protein S
Source: NAR Genom Bioinform. 2022 Aug 22;4(3):lqac058. doi: 10.1093/nargab/lqac058 (PMC9394169; doi:10.1093/nargab/lqac058)
Supplement: lqac058_Supplemental_Files [file lqac058_supplemental_files.zip › Figure S2AG.pdf]

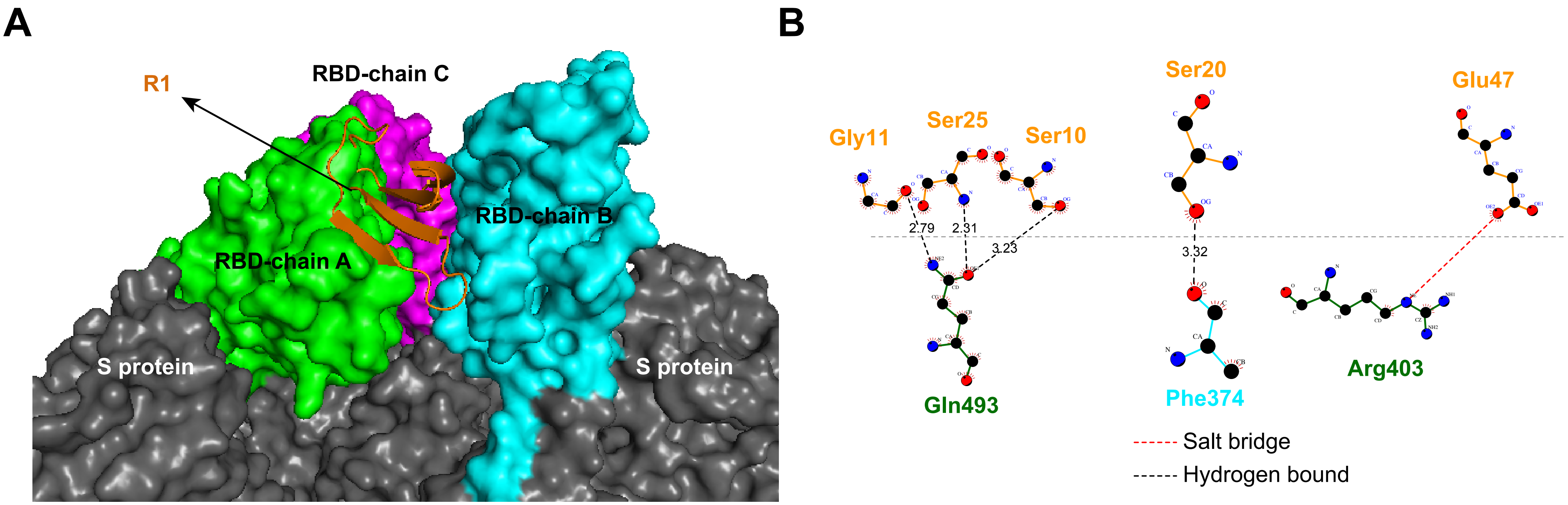
A
R1
RBD-chain C
RBD-chain B
RBD-chain A
S protein
S protein
B
Ser20
Glu47
Ser25
Gly11
Ser10
2.79
2.31
3.23
3.32
Gln493
Phe374
Arg403
Salt bridge
Hydrogen bound
