# Supplementary material for: A computational approach to rapidly design peptides that detect SARS-CoV-2 surface protein S
Source: NAR Genom Bioinform. 2022 Aug 22;4(3):lqac058. doi: 10.1093/nargab/lqac058 (PMC9394169; doi:10.1093/nargab/lqac058)
Supplement: lqac058_Supplemental_Files [file lqac058_supplemental_files.zip › Supplementary Material Legend.docx]

**Supplementary Material Legend**

**Figure S1**. (A) Molecular docking of R1 into the functional trimeric assembly of S protein (grey) reveals target binding between RBD of chain A (green) and chain B (magenta). (B) Five critical amino acid residues of R1 (brown) and their predicted target bindings residues on RBD of chain A (green) and chain B (magenta) as analyzed by LigPlot+, revealing residue spatial distances in angstroms.

**Figure S2**. Salivary peptidomics of SARS-CoV-2, with peptide distributions landscape in patient saliva. (A and B) Average amino acid length of naturally existing SARS-CoV-2 peptides detected by our salivary peptidomics approach for the SARS-CoV-2 Alpha strain (A) or the wild-type SARS-CoV-2 strain, respectively. (C) SARS-CoV-2 peptide landscape detected in patient saliva cover non-structural and structural proteins. Lower panel, protein landscape of SARS-CoV-2 aligned with the detected peaks for comparison.

**Figure S3**. Representative core salivary peptide detected for NSP5 by our MS/MS peptidomics approach. Dimer structure of NSP5 (PDB: 7KHP) with the P9-G15 region detected in patient saliva was found to reside in the dimer interface of NSP5. Region is colored in yellow and red for each monomeric subunit (A), with S10, G11 and E14 deemed critical for NSP5 homodimerization.

**Figure S4**. A representative interaction profile generated by InSiPS. The X- and Y-axes represent the amino acids corresponding to the RBD region and R1 peptide, respectively. Scale represents normalized interaction scores using PIPE (12).
