# Supplementary material for: A computational approach to rapidly design peptides that detect SARS-CoV-2 surface protein S
Source: NAR Genom Bioinform. 2022 Aug 22;4(3):lqac058. doi: 10.1093/nargab/lqac058 (PMC9394169; doi:10.1093/nargab/lqac058)
Supplement: lqac058_Supplemental_Files [file lqac058_supplemental_files.zip › Table S1-Top100-Detected-Peptides.pdf]

Supplementary Table S1: List of 100 original peptides and their SEQUEST scores detected by MS.

Sample	Prey	Peptide Sequence	Peptide Xcorr	Peptide Deltcn
B.1.1.7_Patient_3	NCAP_SARS2	SSRGTSPARMAGNGGDAALALLLLDRLNQLESK	2.3677	0.1322
B.1.1.7_Patient_3	nsp12	FKELLVYAADPAMHAASGNLLL	2.2943	0.1152
B.1.1.7_Patient_3	nsp12	MSYEDQDALFAYTKRNVIPTITQMNLKYA	2.1918	0.1979
B.1.1.7_Patient_3	nsp3	GKPLEFGATSAALQP	2.0685	0.2041
B.1.1.7_Patient_3	nsp3	LMCQPILLLDQALVSDVGDSAEVAVKMFDA	2.037	0.1828
B.1.1.7_Patient_3	nsp3	TNSRIKASMPTTIAKNTVKSVGKFCLEASFN	2.3663	0.164
B.1.1.7_Patient_3	nsp5	VLACYNGSPSGVYQCAMRPNFTIKGSFLNG	2.0828	0.1605
B.1.1.7_Patient_3	SPIKE_SARS2	DEMIAQYTSALLAGTITSGWTFGAGAALQIPFA	2.0603	0.2076
B.1.1.7_Patient_7	NCAP_SARS2	PQRQKKQQTVTLLPAADLDD	2.0414	0.2225
B.1.1.7_Patient_7	nsp12	AMRNAGIVGVLTLDNQDLNGNWYDFGD	2.4239	0.4761
B.1.1.7_Patient_7	nsp12	ANFNVLFSTVFPPTSFGPLVR	2.0312	0.1243
B.1.1.7_Patient_7	nsp12	FNKKDWYDFVENPD	2.1643	0.1166
B.1.1.7_Patient_7	nsp12	GGWHNMLKTVYSDVENPHL	2.1581	0.2709
B.1.1.7_Patient_7	nsp12	GGWHNMLKTVYSDVENPHL	2.2274	0.1462
B.1.1.7_Patient_7	nsp12	PKCDRAMPNMLRIMASLVLARKHTTCCSLSHRFYRLANECAQVL	2.1303	0.166
B.1.1.7_Patient_7	nsp12	VSAARLTPCGTGTSTDVVYRAF	2.5435	0.1095
B.1.1.7_Patient_7	nsp13	CCKCCYDHVISTSHKLVLSVNPYVCNAPGCDVTDVTQLYLGG	2.4444	0.1562
B.1.1.7_Patient_7	nsp13	TVNALPETTADIVVFDEISMA	2.0134	0.2322
B.1.1.7_Patient_7	nsp15	FENKTTLPVNVA	2.0863	0.2196
B.1.1.7_Patient_7	nsp16	LPKGIMMNVAKYTQLCQYLNTLTLA	2.8262	0.3251
B.1.1.7_Patient_7	nsp16	LPKGIMMNVAKYTQLCQYLNTLTLA	2.1628	0.3182
B.1.1.7_Patient_7	nsp16	LPKGIMMNVAKYTQLCQYLNTLTLA	2.1108	0.1999
B.1.1.7_Patient_7	nsp2	ESCGNFKVTKGKAKKGAWNIG	2.074	0.3827
B.1.1.7_Patient_7	nsp3	DLGACIDCSARHINAQVAKSHNIALIWNVKDFMSLSE	2.0927	0.2392
B.1.1.7_Patient_7	nsp3	FNYLKSPNFSKLINIIIWFLLLSVCLGSLIYSTAALGVLMSN	2.0677	0.1099
B.1.1.7_Patient_7	nsp3	LSLQFKRPINPTDQSSYIVDSVTVKN	2.0747	0.137
B.1.1.7_Patient_7	nsp3	QALVSDVGDSAEVAVKMFDA	2.0546	0.1767
B.1.1.7_Patient_7	nsp3	SMPTTIAKNTVKSVGKFCLEASFNYLKSPNFSKLINI	2.5098	0.1403
B.1.1.7_Patient_7	nsp9	CRFVTDTPKGPKVKYLYFI	2.7472	0.1679
B.1.1.7_Patient_7	ORF9B_SARS2	ARKTLNSLEDKAFQLTP	2.1921	0.18
B.1.1.7_Patient_7	SPIKE_SARS2	RTFLLKYNENGTITDAVDCA	2.238	0.3317
B.1.1.7_Patient_7	SPIKE_SARS2	VDLGDISGINASVVNIQKEIDRLNEVAKNLNESLIDLQEL	2.1701	0.1073
B.1.1.7_Patient_7	SPIKE_SARS2	VRDLPQGFSALEPLVDLPIGINITRFQTLLALHRSYL	2.2636	0.1968
B.1.1.7_Patient_7	VME1_SARS2	LNVPLHGTILTRPLLE	2.1898	0.2764
B.1.1.7_Patient_7	Y14_SARS2	VATVQEIQLQAAVGELLLLEWL	2.1147	0.2131
B.1.1.7_Patient_8	NCAP_SARS2	DGKMKDLSPRWYFYYLGTGPEAGLPYGANKDGIIWVA	2.1978	0.1068
B.1.1.7_Patient_8	NCAP_SARS2	SSPDDQIGYYRRATRRIRGGDGKMKDLSPRWYFYYLGT	2.0127	0.1593
B.1.1.7_Patient_8	NCAP_SARS2	SSRGTSPARMAGNGGDAALALLLL	2.3404	0.3121
B.1.1.7_Patient_8	nsp12	ANFNVLFSTVFPPTSFGPLVR	2.6359	0.1751
B.1.1.7_Patient_8	nsp3	LSLQFKRPINPTDQSSYIVDSVTVKN	2.3214	0.313
B.1.1.7_Patient_8	nsp3	LSLQFKRPINPTDQSSYIVDSVTVKN	2.2156	0.2013
B.1.1.7_Patient_8	nsp3	LSLQFKRPINPTDQSSYIVDSVTVKN	2.4787	0.1112
B.1.1.7_Patient_8	nsp6	LLVLVQSTQWSLFFFLYENAFLPFAMGIIAMSAFAMMFVKH	2.0933	0.1965
B.1.1.7_Patient_8	nsp6	QEFRYMNSQGLLPPKNSIDAFKLNIKLLGVGGKPCIKVA	2.1314	0.2078
B.1.1.7_Patient_8	SPIKE_SARS2	FTVEKGIYQTSNFRVQPT	2.0438	0.2313
B.1.1.7_Patient_8	SPIKE_SARS2	NNCTFEYVSQPFLMDLEGKQGNFKN	2.5515	0.2064
B.1.1.7_Patient_8	SPIKE_SARS2	NRKRISNCVADYSVLYNSASFSTFKCYGVSPTKLND	2.1707	0.1252
Wuhan_Patient_2	nsp3	GYTVEEAKTVLKKCKSAFY	2.4413	0.1575
Wuhan_Patient_4	nsp1	EKGVLPQLEQPYVFIKRSDARTAPHGHVMVELVAELEGIQYGRSGETLGVLVP	2.5407	0.1011
Wuhan_Patient_4	nsp12	GNKIADKYVRNLQHRLYECLYRNRDVDTDFVNEFYAYLRKHFSMMILSDDAVVCFNST	2.4327	0.1179
Wuhan_Patient_5	nsp12	AALTNNVAFQTVKPGNFNKDFYDFAVSKGFFKEGSSVELKH	2.3188	0.107
Wuhan_Patient_5	nsp13	ARVECFDKFKVNSTLEQYVFCTVNALPETTADIVVFDEI	2.1702	0.1474
Wuhan_Patient_5	nsp13	CANGQVFGLYKNTCVGSDNVTDFNAIATCDWTNAGDYILANT	3.2674	0.2079
Wuhan_Patient_5	nsp14	IEYPIIGDELKINAACRKVQHMVVKAALLADKFPVLHDIGN	3.372	0.1855
Wuhan_Patient_5	nsp2	AFKQIVESCGNFKVTKGKAKKGAWNIGEQKSILSPLYAFASE	2.2439	0.1268
Wuhan_Patient_5	nsp3	FSGYLKLTDNVYIKNADIVEEAKKVKPTVVVNAANVYLKHGG	3.4025	0.1433
Wuhan_Patient_5	nsp3	FYVLGLAAIMQLFFSYFAVHFISNSWLMWLIINLVQMA	2.75	0.2599
Wuhan_Patient_5	nsp3	LDNLRANNTKGSLPINVIVFDGKSKCEESSAKSASVYYSQLM	2.6304	0.1201
Wuhan_Patient_5	nsp3	NTKGSLPINVIVFDGKSKCEESSAKSASVYYSQLMCQPILLL	2.6026	0.2545
Wuhan_Patient_5	nsp4	CYTPSKLIEYTDFATSACVLAAECTIFKDASGKPVPYCYDT	2.4811	0.2026
Wuhan_Patient_5	nsp5	IQPGQTFSVLACYNGSPSGVYQCAMRPNFTIKGSFLNGSCGS	3.0017	0.235
Wuhan_Patient_5	nsp6	TWLDMVDTSLSGFKLKDCVMYASAVVLLILMTARTVYDDG	2.7194	0.1135
Wuhan_Patient_5	nsp8	MADQAMTQMYKQARSEDKRAKVTSAMQTMLFTMLRKLDN	3.0853	0.1479
Wuhan_Patient_6	AP3A_SARS2	YLYALVYFLQSINFVRIIMRLWLC	2.0292	0.1213
Wuhan_Patient_6	NCAP_SARS2	TFPPTEPKKDKKKKADETQALPQRQKKQQTVTLLPAADLD	2.3386	0.1331
Wuhan_Patient_6	nsp12	ERFVSLAIDAYPLTKHPNQEYADVFHL	2.199	0.1001
Wuhan_Patient_6	nsp12	RNAGIVGVLTLDNQDLNGNWYDFGDFIQTTPGSGVPVV	2.4214	0.1348
Wuhan_Patient_6	nsp13	CANGQVFGLYKNTCVGSDNVTDFNAIATCDWTNAGDYILANT	3.4867	0.1473
Wuhan_Patient_6	nsp13	CANGQVFGLYKNTCVGSDNVTDFNAIATCDWTNAGDYILANT	2.9001	0.1288
Wuhan_Patient_6	nsp16	FFTYICGFIQQKLALGGSVAIKITEHSWNADLYKLMGHFA	2.0585	0.1157
Wuhan_Patient_6	nsp2	GTENLTKEGATTCGYL	2.1486	0.3014
Wuhan_Patient_6	nsp2	KKLDGFMGRIRSVYPVASPNECNQMCLSTLMKCD	2.2297	0.1131
Wuhan_Patient_6	nsp2	PRVEKKKLDGFMGRIRSVYPVASPNECNQMCLSTLMKCD	2.0403	0.3343
Wuhan_Patient_6	nsp3	AVFDKNLYDKLVSSFLEMKSEKQVEQKIAEIPKEEVKPF	2.1853	0.1311
Wuhan_Patient_6	nsp3	DVVQEGVLTAVVIPT	2.0348	0.1887
Wuhan_Patient_6	nsp3	KVFTTVDNINLHTQVVDMSM	2.1896	0.1445
Wuhan_Patient_6	nsp3	KVLNEKCSAYTVELGTEVNEFA	2.0056	0.2336
Wuhan_Patient_6	nsp3	LPSIISNEKQEILGTVSWNLREMLAHAEETRKLMPVCVETKAIVSTIQRKYK	2.3648	0.285
Wuhan_Patient_6	nsp3	LREVRTIKVFTTVDNINLHTQVVDMSMTYGQQFGPTYLDG	2.7457	0.1865
Wuhan_Patient_6	nsp3	NDLNETLVT	2.0902	0.2372
Wuhan_Patient_6	nsp3	NNAMQVESDDYIATNGPLKVGGSCVLSGHNLAKHCLHVVGPN	2.1702	0.1848
Wuhan_Patient_6	nsp3	QALVSDVGDSAEVAVKMFDAYVNTFSST	2.2162	0.1257
Wuhan_Patient_6	nsp3	QQESPFVMMSAPPAQYELK	2.3473	0.1057
Wuhan_Patient_6	nsp3	VFTTVDNINLHTQVV	2.2806	0.1199
Wuhan_Patient_6	nsp3	YILFTRFFYVLGLAA	2.1812	0.3136
Wuhan_Patient_6	nsp5	FVRIQPGQTFSVLAC	2.2845	0.2982
Wuhan_Patient_6	nsp5	GDRWFLNRFTTTLNDFNLVAMKYNYEPLTQDHVDILGPLSAQTGIAVLDMCASL	2.0559	0.1414
Wuhan_Patient_6	nsp5	HVDILGPLSAQTGIAV	2.0268	0.1895
Wuhan_Patient_6	nsp5	IQPGQTFSVLACYNGSPSGVYQCAMRPNFTIKGSFLNGSCGS	2.8779	0.1209
Wuhan_Patient_6	nsp6	FAMMFVKHKHAFLCLFLLPSLATVAY	2.1937	0.2228
Wuhan_Patient_6	nsp6	LVSTQEFRYMNSQGLLPP	2.1682	0.1661
Wuhan_Patient_6	nsp6	QEFRYMNSQGLLPPKNSIDAFKLNIKLLGVGGKPCIKVATVQ	2.4019	0.2016
Wuhan_Patient_6	nsp6	VLLILMTARTVYDDGARRVWTLMNVLTLVYKVYYGNALDQAISMWALIISVT	2.2672	0.1263
Wuhan_Patient_6	nsp8	MQTMLFTMLRKLDNDALNNIIN	2.0229	0.1025
Wuhan_Patient_6	nsp8	SEFSSLPSYAAFAT	2.2938	0.3565
Wuhan_Patient_6	ORF9B_SARS2	RLGSPLSLNMARKTLNSLEDKAFQLTPI	2.068	0.2801
Wuhan_Patient_6	SPIKE_SARS2	GAAAYYVGYLQPRTFLLKYN	2.1589	0.3219
Wuhan_Patient_6	SPIKE_SARS2	SPRRARSVASQSIIAYTMSLGA	2.0204	0.2192
Wuhan_Patient_6	VEMP_SARS2	KNLNSSRVPDLLV	2.4534	0.2198
Wuhan_Patient_6	VEMP_SARS2	KNLNSSRVPDLLV	2.0903	0.1036
