# Supplementary figures and images for: A computational approach to rapidly design peptides that detect SARS-CoV-2 surface protein S
Source: NAR Genom Bioinform. 2022 Aug 22;4(3):lqac058. doi: 10.1093/nargab/lqac058 (PMC9394169; doi:10.1093/nargab/lqac058)

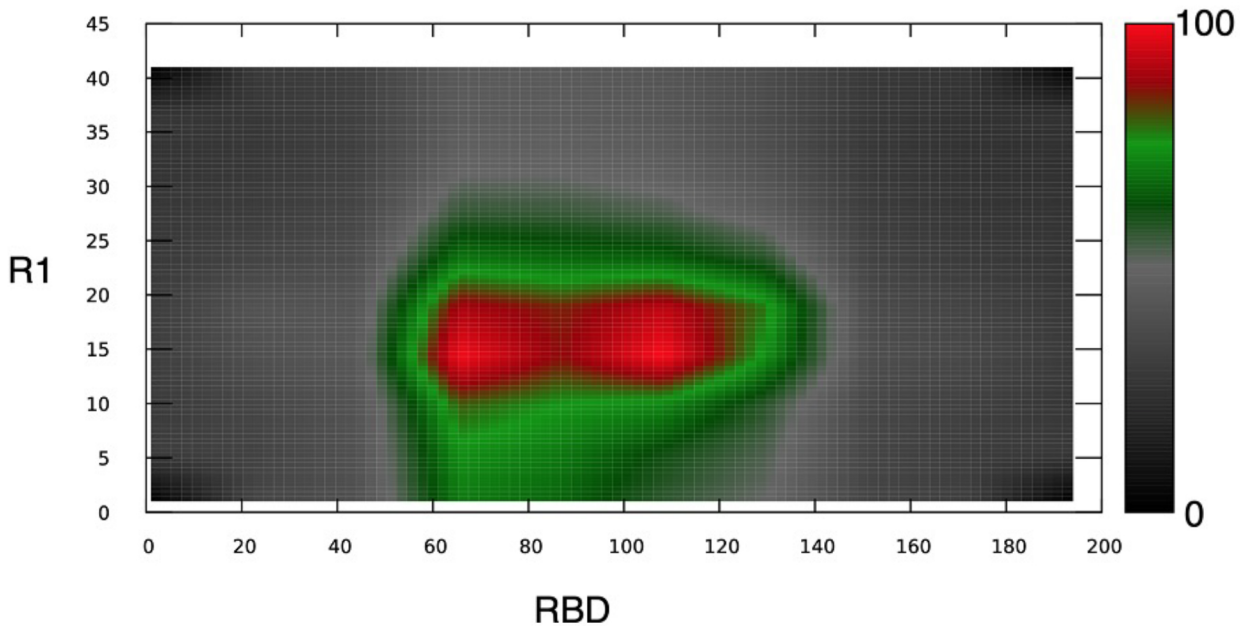

Supplement: lqac058_Supplemental_Files [file lqac058_supplemental_files.zip › Figure S1AG.pdf]

**A**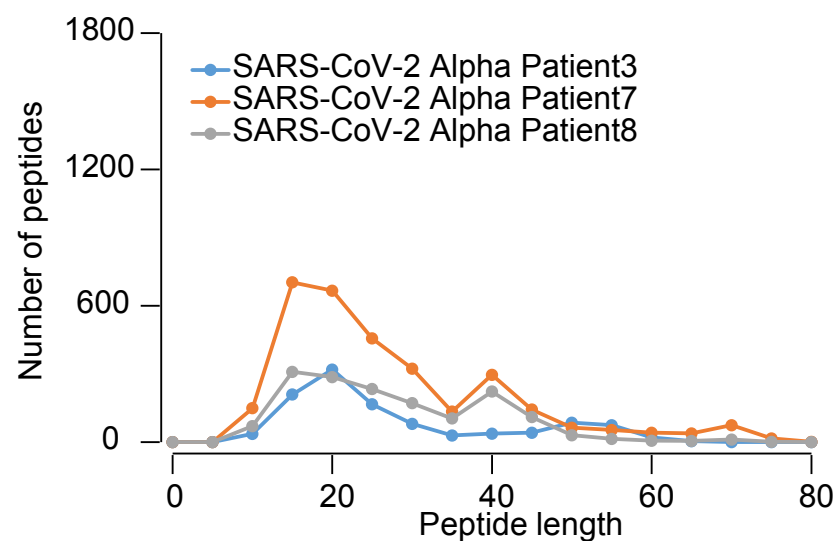**B**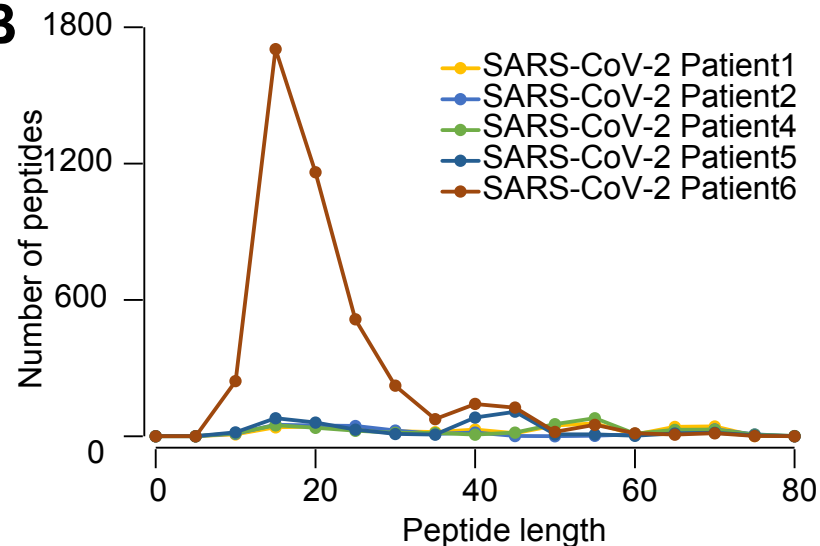**C**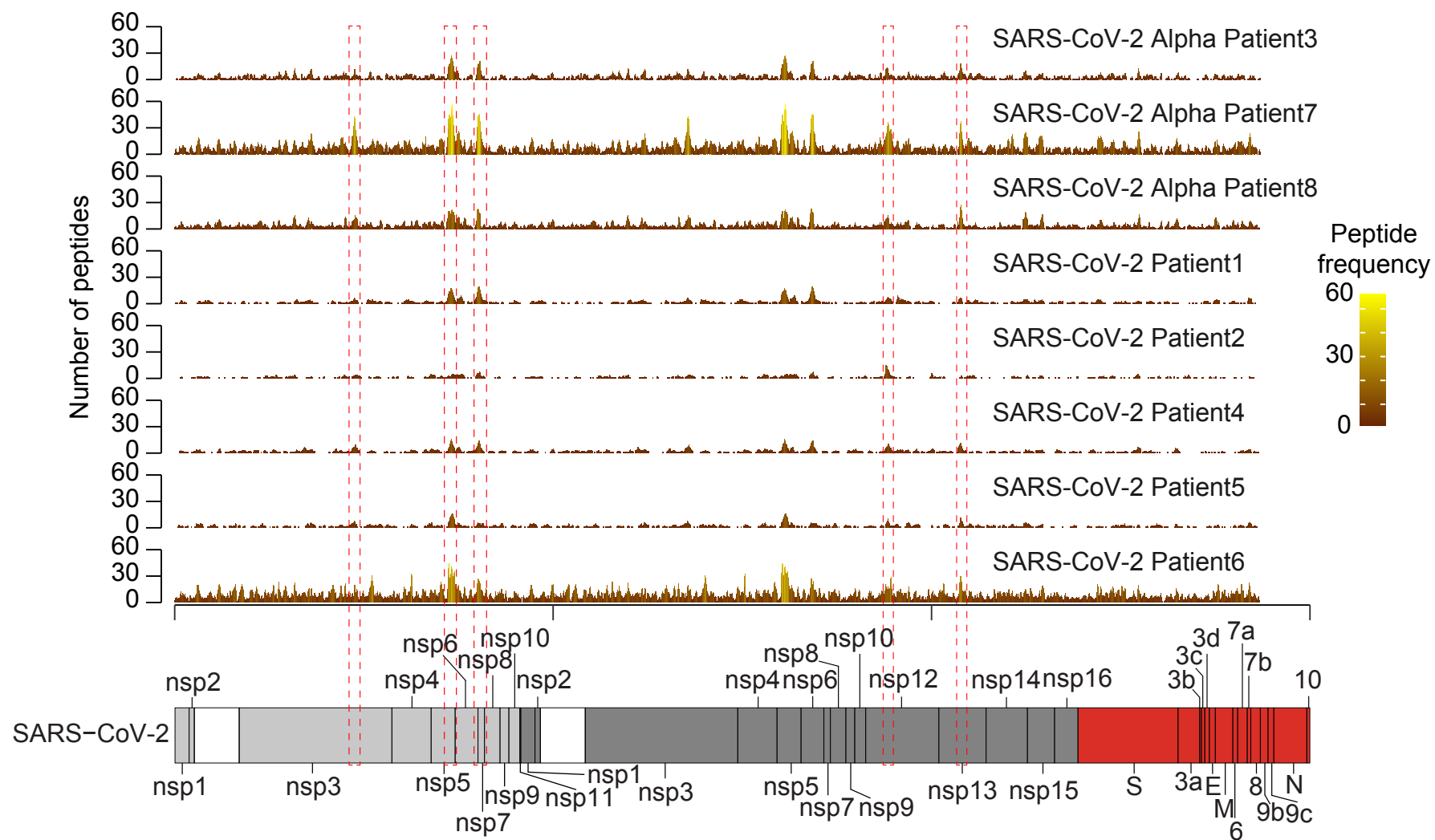

Supplement: lqac058_Supplemental_Files [file lqac058_supplemental_files.zip › Figure S3AG.pdf]

**A**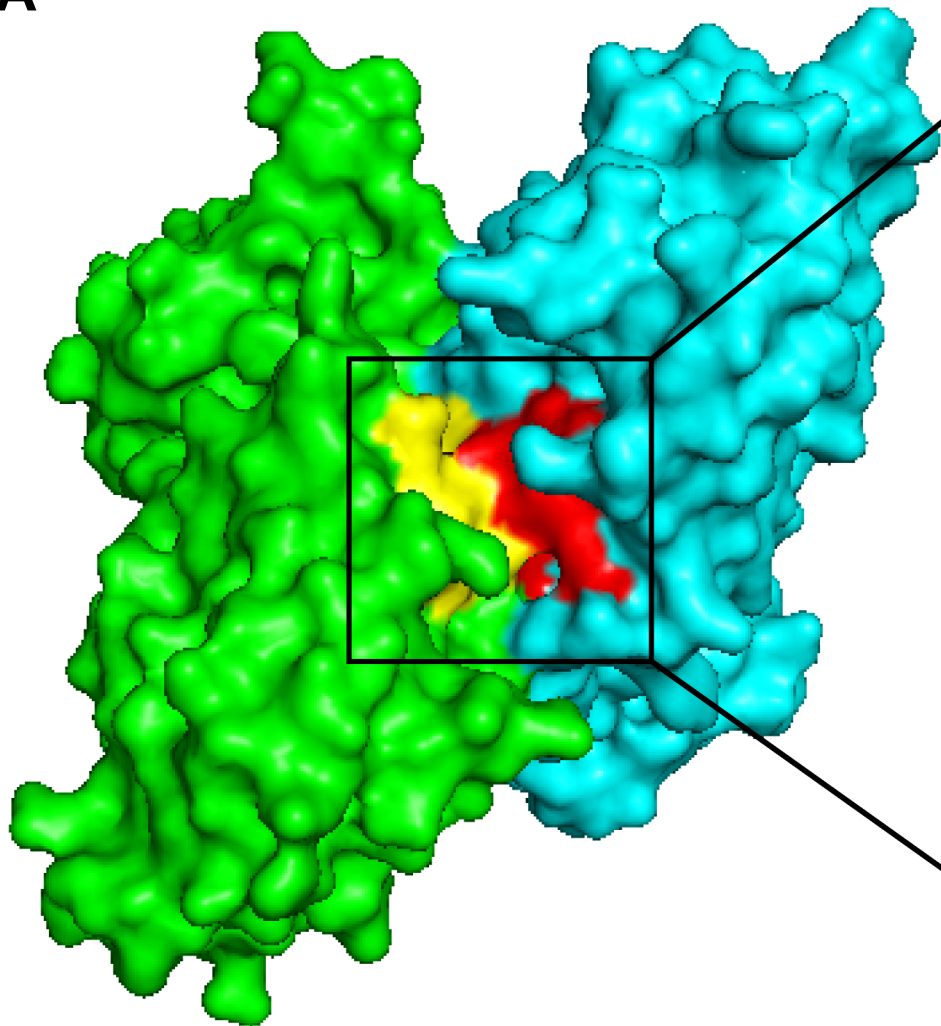**B**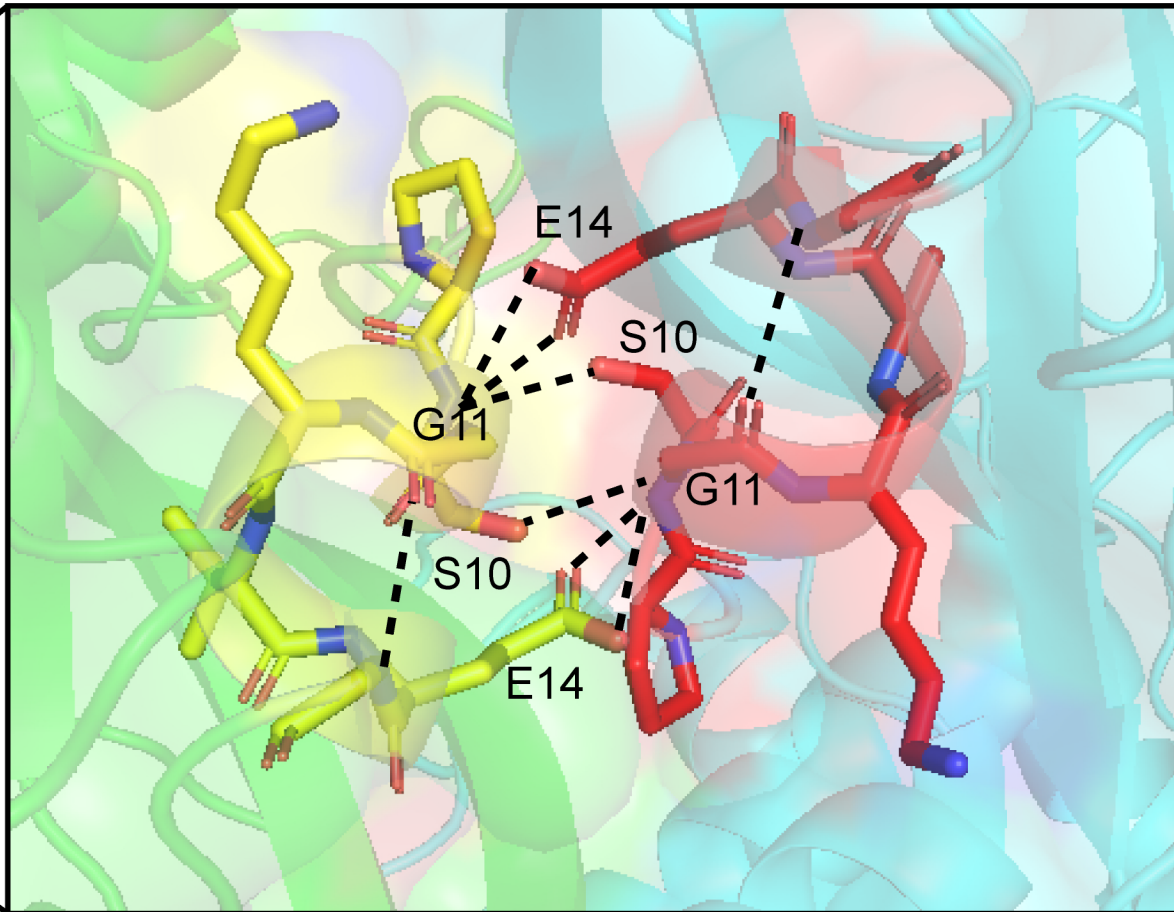

Supplement: lqac058_Supplemental_Files [file lqac058_supplemental_files.zip › Figure S4AG.pdf]
